# Differences in nitrogen and phosphorus sinks between the harvest and non-harvest of *Miscanthus lutarioriparius* in the Dongting Lake wetlands

**DOI:** 10.3389/fpls.2022.989931

**Published:** 2022-09-08

**Authors:** Zenghui Peng, Yuhang Du, Shiyu Niu, Lianlian Xi, Yandong Niu, Youzhi Li

**Affiliations:** ^1^College of Resources and Environment, Hunan Agricultural University, Changsha, China; ^2^Hunan Provincial Key Laboratory of Rural Ecosystem Health in Dongting Lake Area, Hunan Agricultural University, Changsha, China; ^3^Hunan Academy of Forestry, Changsha, China; ^4^Hunan Dongting Lake Wetland Ecosystem National Positioning Observation and Research Station, Yueyang, China

**Keywords:** nitrogen sinks, phosphorus sinks, harvest, non-harvest, litter decomposition, relative release indices

## Abstract

Plant non-harvest changes element circulation and has a marked effect on element sinks in the ecosystem. In this study, a field investigation was conducted on the fixation of nitrogen and phosphorus in *Miscanthus lutarioriparius*, the most dominant plant species in the Dongting Lake wetlands. Further, to quantitatively compare the difference in nitrogen and phosphorus sinks between harvest and non-harvest, an *in situ* experiment on the release of the two elements from two types of litters (leaves and stems) was studied. The nitrogen concentrations in the plant had no significant relationship with the environmental parameters. The phosphorus concentrations were positively related to the plot elevation, soil organic matter, and soil total potassium and were negatively related to the soil moisture. The leaves demonstrated a higher decomposition coefficient than that of the stems in the *in situ* experiment. The half decomposition time was 0.61 years for leaves and 1.12 years for stems, and the complete decomposition time was 2.83 years for leaves and 4.95 years for stems. Except for the nitrogen concentration in the leaves, all the concentrations increased during the flood period. All concentrations unsteadily changed in the backwater period. Similarly, except for the relative release index of nitrogen in the leaves, all the relative release indices decreased in the flood period. At the end of the *in situ* decomposition experiment, the relative release indices of both the nitrogen and phosphors were greater than zero, indicating that there was a net release of nitrogen and phosphorus. Under the harvest scenario, the aboveground parts of the plant were harvested and moved from the wetlands, thus increasing the nitrogen and phosphorus sinks linearly over time. The fixed nitrogen and phosphorus in the aboveground parts were released under the non-harvest scenario, gradually accumulating the nitrogen and phosphorus sinks from the first year to the fifth year after non-harvest, reaching a maximum value after the fifth year. This study showed that the nitrogen and phosphorus sinks greatly decreased after the non-harvest of *M. lutarioriparius* compared to that after harvest. It is recommended to continue harvesting the plant for enhancing the capacity of element sinks.

## Introduction

A large amount of nitrogen and phosphorus has been released into the environment in the past several decades, causing great threats to the ecosystem (Grizzetti et al., 2020). Approximately 605 million moles of nitrogen and 36 million moles of phosphorus are reportedly inputted into the Narragansett Bay each year, eventually getting deposited in the sediment (Nixon et al., 2008). Recently, the nitrogen and phosphorus concentrations in the sediments of the major rivers of China (such as the Yangtze River, Yellow River, and Huai River) were found to be much higher than the soil background values (Yang et al., 2017). This nitrogen and phosphorus will be released from the sediments again and decrease the water quality and accelerate eutrophication (Jin et al., 2006). Due to their important roles in element sinks, plant species such as *Pistia stratiotes*, *Eichhornia crassipes*, and *Phragmites australis* are commonly used to remove the nitrogen and phosphorus from aquatic ecosystems (Lu et al., 2010; Nash et al., 2019; Rezania et al., 2019).

For plants, the capacity of nitrogen and phosphorus sinks depends on the amount of elements fixed in the plant. Nitrogen and phosphorus fixation is a biological process and is associated with certain environmental factors. Studies have shown soil pH to be one of the main factors affecting the fixation of phosphorus (Barrow, 2017). In high soil moisture conditions, low oxygen availability limits nitrogen fixation (Sajedi et al., 2012). Under the comprehensive influence of environmental factors, plant organs exhibit different nitrogen and phosphorus allocation patterns. For example, compared with stems and roots, the leaves in 30 species of desert plants demonstrated higher nitrogen and phosphorus concentrations (Luo et al., 2021). Unlike terrestrial plants, aquatic plants can obtain nitrogen and phosphorus from both soil and water, thus demonstrating higher element concentrations in aboveground parts than in underground parts (Granéli and Solander, 1988). Detailed studies on nitrogen and phosphorus fixation are therefore necessary for quantitatively evaluating the capacity of element sinks.

The fixed nitrogen and phosphorus in plants is released into the environment with the decomposition of litter. The release of nitrogen and phosphorus from litter is also a biological process, and the amount of the released elements can be reflected by the weight of residual litter and elemental concentrations (van der Valk et al., 1991; Kuehn and Suberkropp, 1998). The negative exponential decay model is usually adopted for estimating the mass of residual litters in the decomposition process (Olson, 1963). The nitrogen and phosphorus concentrations in litters show a dynamic balance between element release from litters into the environment and element fixation by litters from the environment (Zhang et al., 2020). A study conducted on the decomposition of forest litter showed that nitrogen concentration in litters increased in the first and second years of the decomposition and decreased in the third year (McClaugherty et al., 1985). Studies on the release process of nitrogen and phosphorus in litter decomposition are therefore also necessary for quantitatively evaluating the capacity of element sinks.

Dongting Lake is the second largest freshwater lake in China and is connected to the Yangtze River and the “four rivers” of Hunan (Xiang, Zi, Yuan, and Li rivers). In the catchment, a large amount of nitrogen, phosphorus, and heavy metals are imported into the lake and deposited in the sediments. *Miscanthus lutarioriparius*, a perennial, emergent macrophyte, is distributed on the beach of the lake and is the most dominant species in this area (Xu et al., 2021). In the past several decades, the aboveground parts of this plant have been harvested as a raw material for papermaking, causing the removal of a great number of elements from the lake (Yao et al., 2018). However, it is now forbidden to harvest *M. lutarioriparius* owing to the important role of the lake in biodiversity protection and the disappearance of papermaking industries in the region. Typically, the aboveground parts of *M. lutarioriparius* will decompose, and the fixed elements will be released into aquatic ecosystems. The non-harvest of the plant, therefore, evokes high concerns regarding the potential ecological risk caused by the possible decline in element sinks. Few studies have quantitatively estimated the difference in element sink between plant harvest and non-harvest to date (Morton et al., 2010). In this context, harvest refers to harvesting the aboveground parts of plants once a year, while non-harvest means removing human disturbance and letting plants fall and decompose in the wild.

To this end, a field investigation and an *in situ* experiment were conducted to explore the fixation of nitrogen and phosphorus in the aboveground parts of the plants and the release of the two elements to reveal the changes in nitrogen and phosphorus sinks after the policy change from harvest to non-harvest.

## Materials and methods

### Study area

Dongting Lake has an area of 2,625 km^2^ (28(382gt98452gting15402gti30102gting Lake has an area of 2n a by floods from the Yangtze, Xiang, Zi, Yuan, and Li rivers. The annual fluctuation in the water level is approximately 12–14 m, with the maxima in July–August and minima in January–February (Xie et al., 2015). Since the 1960s, *M. lutarioriparius* was planted in Dongting Lake for paper manufacture. This plant generally germinates in March–April and is harvested during November–December. The total distribution area of *M. lutarioriparius* in the lake is approximately 585 km^2^ and accounts for 22.3% of the total area of Dongting Lake (Xu et al., 2021). Currently, the lake is a global hotspot for biodiversity conservation, with three international important wetlands and two national wetland nature reserves.

### Field investigation on nitrogen and phosphorus fixation

**FIGURE 1:**
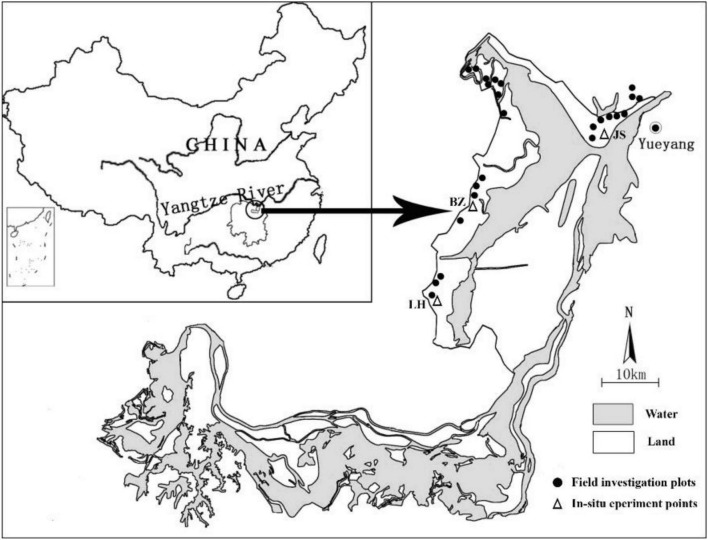
The field investigation plots (circles) and *in situ* experiment points (triangles; JS: Junshan; BZ: Beizhouzi; LH: Luhu) in the Dongting Lake wetlands.

In November 2016, 24 vegetated plots were selected on the beach of Dongting Lake (Figure 1). The geographical information of each plot was recorded using a hand-held GPS (UniStrong, China). A quadrat (1 m × 1 m) was chosen for investigation in each vegetated plot. The stems in each sampled quadrat were cut from the soil surface and cut into pieces, and the leaves were collected from the stems. The roots were dug from a soil depth of 0–30 cm and carefully washed such that they were free of soil. The mixed soil samples of 0–30 cm were collected for determining the environmental parameters in the soil. These vegetation and soil samples were sealed in plastic bags and brought back to the laboratory for further analysis.

### *In situ* experiment on nitrogen and phosphorus release

**FIGURE 2:**
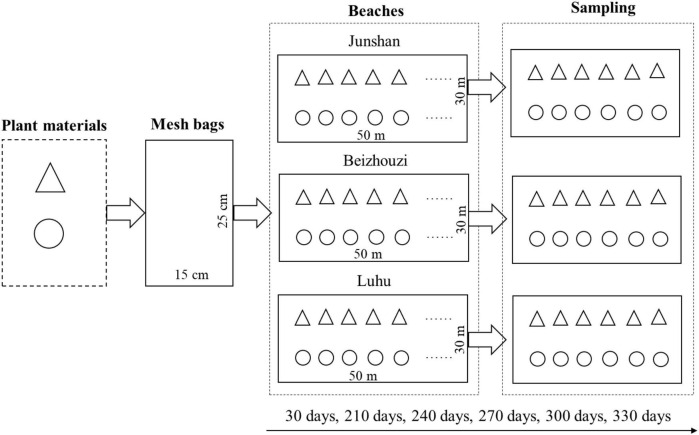
Flow picture of the implementation of the *in situ* experiment (triangles, leaves; circles, stems).

In November 2019, the leaves and stems of *M. lutarioriparius* were collected from the beach of Dongting Lake and air-dried. In April 2020, 5 g each of leaves and stems were put into separate nylon mesh bags (15 cm × 25 cm) with 1 mm apertures. These bags were taken into *M. lutarioriparius* communities on three beaches (Junshan, Beizhouzi, and Luhu) of Dongting Lake. In each plant community, 72 bags, including 36 leaf bags and 36 stem bags, were placed on the soil surface. These bags were placed in the center of the plant communities with an area of 30 width and 50 m length; the distance between bags was 1 m. After the plant bags were put into the plant communities for 30, 210, 240, 270, 300, and 330 days, the samples were taken with six replicates and brought back to the laboratory for further analysis (Figure 2).

### Measurements of biomass, and nitrogen and phosphorus concentrations in plants

In the field investigation and *in situ* experiment, samples of roots, stems, and leaves were dried separately in an oven at 80°C for 48 h before measuring their dry weight. The biomass of plant organs was defined as dry mass per square meter, and plant total biomass was calculated as the total weight of roots, stems, and leaves. Plant organs were ground to a fine powder and passed through a 0.15 mm mesh. Material less than 0.15 mm was then used to test the concentrations of nitrogen and phosphorus. The nitrogen concentrations in plants were analyzed using a continuous flow analyzer (Auto Analyzer 3 HR, Seal Analytical, Germany), and phosphorus concentrations were measured by the molybdenum-antimony anti-colorimetric method (NY/T 2017-2011).

### Measurement of soil properties

In the field investigation, soil properties were measured according to the Chinese national standards (Liu, 1996). Soil moisture was calculated as [(FW - DW)/FW] × 100%, where FW is the soil fresh weight and DW is the soil dry weight after drying in an oven at 105°C for 48 h. The remaining fresh soil was air-dried in the shade for the analysis of soil variables. The pH, electrolyte leakage, organic matter concentration, total nitrogen concentration, alkali-hydrolyzable nitrogen concentration, total phosphorus concentration, available phosphorus concentration, and total potassium concentration were then measured (Yao et al., 2018).

### Data calculation

The amount of nitrogen or phosphorus fixed by a plant organ was calculated using the following equation:

$$E_{f}=M\times C,$$

where *E*_f_ is the amount of an element (nitrogen or phosphorus) fixed in the plant organ (leaf, stem, or root), *M* is the dry mass of the plant organ, and *C* is the concentration of an element in the organ. The amount of elements fixed by the aboveground parts of plants was the sum of the amount of the elements fixed by leaves and stems.

The mass residual ratio of litter (leaf or stem) was calculated using the following equation:

$$R=\frac{M_{t}}{M_{0}}\times 100\%,$$

where *R* is the mass residual ratio of litter, *M*_t_ is the litter mass after decomposing time *t*, and *M*_0_ is the initial litter mass.

A negative exponential decay model was used to analyze the mass residual ratio of litter in litter decomposition (Olson, 1963):

$$y=a\,e^{-k\,t},$$

where *y* is the mass residual ratio of litter, *a* is the fitting parameter, *k* is the annual decomposition coefficient, and *t* is the decomposing time.

**FIGURE 3:**
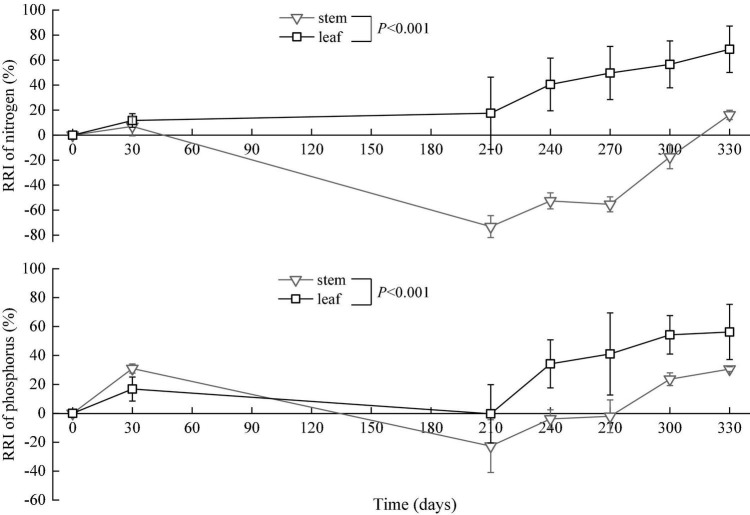
Dynamics of relative release indices (RRI, means ± SD) of nitrogen and phosphorus in the *in situ* experiment.

The amount of an element released in litter decomposition (*E*_r_) and the relative release index (*RRI*) were calculated using the following equations:

$$E_{r}=M_{0}\times C_{0}-M_{t}\times C_{t}$$

$$R\,R\,I=\frac{M_{0}\times C_{0}-M_{t}\times C_{t}}{M_{0}\times C_{0}}\times 100\%,$$

where *M*_0_ is the initial litter mass, *C*_0_ is the initial concentration of an element in the litter, *M*_t_ is litter mass after decomposing time *t*, and *C*_t_ is the concentration of an element in the litter at time *t*. The litter on the 360th day could not be collected due to uncontrollable factors in the field, and thus, the *RRI* in the time was calculated according to the regression model of *RRI* in the leaves and stems (Figure 3).

Nitrogen and phosphorus sinks were evaluated for two scenarios. If the plant is harvested, the annual element sinks were considered equal to *E*_f_. If the plant is not harvested, the element sinks were calculated as follows:

$$S_{n}=\sum_{i=1}^{n}E_{r\,l}+\sum_{i=1}^{m}E_{r\,s},$$

where *S*_n_ is the element sink in the non-harvest scenario; *E*_rl_ and *E*_rs_ are the element contents in residual leaves and stems after a year’s litter decomposition, respectively; and *n* and *m* are the complete decomposition times of leaves and stems, respectively. Considering that the *in situ* experiment was conducted in 1 year, the amounts of both elements in the residual litters in the following years (the second year, third year, etc.) were estimated as the mean element concentrations in the *in situ* experiment of 1 year multiplied by the weight of residual litters calculated with the exponential decay model of the residual litters (y = ae^–kt^).

### Statistical analysis

Multiple comparisons were conducted to test the difference in the biomass, concentrations of nitrogen and phosphorus in plants, and amount of nitrogen and phosphorus fixed in plants using Tukey’s test at the 0.05 significance level. Linear regression analysis between mean concentrations of nitrogen and phosphorus in plants and environmental parameters was performed at the 0.05 significance level. Before analysis, the homogeneity of variances was tested using Levene’s test, and data were log_10_-transformed when necessary to reduce the heterogeneity of variances. The statistical analyses were performed using SPSS Statistics 23.0 (IBM Corp., United States) software, and figures were produced using OriginPro 2021 (OriginLab Corp., United States) software.

## Results

### Concentrations and amount of nitrogen and phosphorus in plants in the field investigation

The concentrations and amounts of nitrogen and phosphorus were significantly different in the three plant organs (Table 1). For concentrations, both nitrogen and phosphorus were the highest in the leaves (16.27 and 1.34 g kg^–1^ for nitrogen and phosphorus, respectively), intermediate in the roots (7.13 and 1.03 g kg^–1^, respectively), and lowest in the stems (3.83 and 0.44 g kg^–1^, respectively). However, the amounts of fixed nitrogen and phosphorus were the highest in the roots (9.42 and 1.55 g m^2^ for nitrogen and phosphorus, respectively), intermediate in the stems (4.46 and 0.49 g m^2^, respectively), and lowest in the leaves (2.66 and 0.21 g m^2^, respectively).

**TABLE 1:** Plant biomass, concentrations, and amounts of nitrogen and phosphorus (means ± SD) fixed in *M. lutarioriparius* in the field investigation.

Plant organs	Plant biomass (g m^–2^)	Element concentrations (g kg^–1^)		Amount of elements (g m^–2^)	
		Nitrogen	Phosphorus	Nitrogen	Phosphorus
Leaf	148.1 ± 102.4^a^	16.27 ± 5.12^c^	1.34 ± 0.32^c^	2.66 ± 2.52^a^	0.21 ± 0.16^a^
Stem	1139.9 ± 467.7^b^	3.83 ± 1.39^a^	0.44 ± 0.20^a^	4.46 ± 2.79^b^	0.49 ± 0.27^b^
Root	1428.7 ± 801.9^c^	7.13 ± 1.56^b^	1.03 ± 0.24^b^	9.42 ± 4.42^c^	1.55 ± 1.03^c^

Different letters in the same column indicate significant differences among plant organs at *P* < 0.05 based on Tukey’s test.

The total amounts of elements fixed by plants were 16.544 g m^2^ for nitrogen and 2.239 g m^2^ for phosphorus. Combined with the area of *M. lutarioriparius* in the Dongting Lake wetlands, approximately 9,680 t nitrogen and 1,310 t phosphorus were fixed by this plant per year, and 4,168 t nitrogen and 404 t phosphorus were fixed by the aboveground parts.

### The relationship between nitrogen and phosphorus concentrations in plants and environmental parameters in the field investigation

The mean nitrogen concentrations in the plant did not exhibit a significant relationship with the studied parameters (Table 2). The mean phosphorus concentrations in the plant were positive related to the plot elevation, soil organic matter, and soil total potassium, and were negatively correlated to the soil moisture.

**TABLE 2:** Linear regression analysis of nitrogen (N) and phosphorus (P) concentrations in *M. lutarioriparius* and environmental parameters in the field investigation.

Environmental parameters	N concentration		P concentration	
	Unstandardized coefficients	*t*-Statistic	Unstandardized coefficients	*t*-Statistic
C	4.720	0.158	–1.27	–0.404
PE	–0.292	–1.186	0.068	2.631*
SM	0.08	0.444	–0.045	–2.369*
SpH	2.237	0.603	–0.018	–0.047
SEL	–0.00008	–0.01	–0.001	–1.564
SOM	–0.091	–0.473	0.049	2.41*
SAN	–0.011	–0.214	–0.01	–1.88
STN	3.749	0.637	0.384	0.622
SAP	0.172	0.884	–0.01	–0.471
STP	–13.292	–1.53	0.329	0.361
STK	–0.192	–0.772	0.057	2.197*
*R* ^2^	0.308		0.599	
*P*	0.805		0.131	

*P < 0.05. PE, plot elevation; SM, soil moisture; SpH, soil pH; SEL, soil electrolyte leakage; SOM, soil organic matter; SAN, soil alkali–hydrolyzable nitrogen; STN, soil total nitrogen; SAP, soil available phosphorus; STP, soil total phosphorus; STK, soil total potassium.

Bold values indicate significant correlations.

### Mass residual ratio of leaves and stems in the *in situ* experiment

**FIGURE 4:**
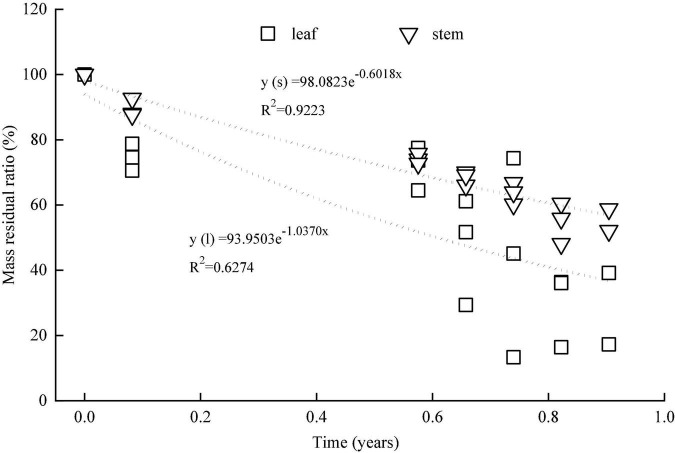
Dynamics of mass residual ratio in the leaves and stems in the *in situ* experiment.

There was an obvious difference in the mass residual ratio between leaves and stems (Figure 4). Over time, the mass residual ratio of the leaves and stems demonstrated negative exponential decay models. The decomposition coefficient was 1.037 for stems and 0.6018 for leaves. Based on the negative exponential decay model, it may be deduced that the half decomposition (50% decomposition) time was 0.61 years for leaves and 1.12 years for stems, and the complete decomposition (95% decomposition) time was 2.83 years for leaves and 4.95 years for stems.

### Release of nitrogen and phosphorus in leaves and stems in the *in situ* experiment

**FIGURE 5:**
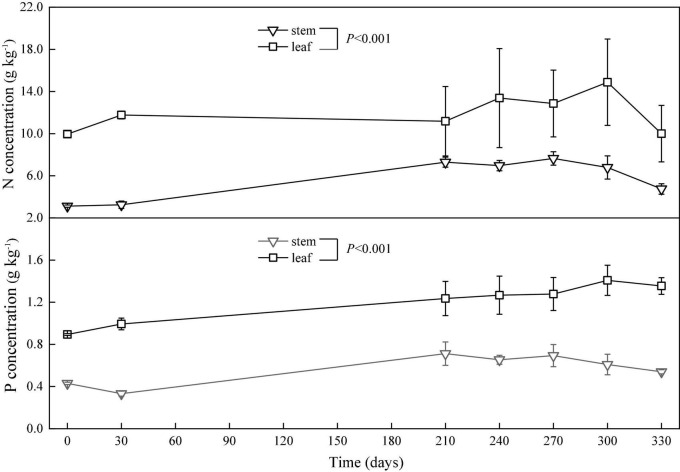
Concentrations (means ± SD) of nitrogen (N) and phosphorus (P) in the leaves and stems in the *in situ* experiment.

The concentrations of nitrogen and phosphorus in the leaves and stems changed with time gradually. The nitrogen concentration in the leaves increased in the period from the 0th to the 30th day, decreased from the 30th to the 210th day, and unsteadily changed from the 210th to the 330th day. However, the phosphorus concentration in the leaves increased from the 0th to the 300th day and decreased from the 300th to the 330th day. Overall, the concentrations of nitrogen and phosphorus in the stems increased from the 0th to the 270th day and decreased from the 270th to the 330th day (Figure 5).

The relative release index of nitrogen in the leaves gradually increased in the period from the 0th to the 330th day. However, the relative release index of nitrogen in the stems increased in the period from the 0th to the 30th day, decreased from the 30th to the 210th day (flood period), and then increased from the 210th to the 330th day (backwater period). The relative release index of phosphorus in the leaves and stems increased in the period from the 0th to the 30th day, decreased from the 30th to the 210th day, and then increased from the 210th to the 330th day. At the end of the *in situ* decomposition experiment, the relative release indices of nitrogen and phosphors were greater than zero (Figure 3).

### Nitrogen and phosphorus sinks in the scenario of harvest and non-harvest of the plant

**FIGURE 6:**
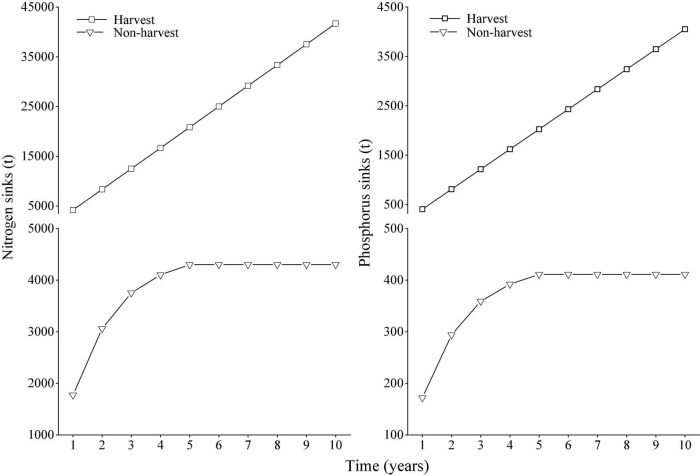
Nitrogen and phosphorus sinks in the scenarios of harvest and non-harvest of *M. lutarioriparius* in the Dongting Lake wetlands over time.

In the scenario of the harvest of the plant, the nitrogen and phosphorus sinks increased linearly over time (Figure 6). In the scenario of non-harvest, the nitrogen and phosphorus sinks gradually increased during the period from the first year to the fifth year after non-harvest. However, after the fifth year of non-harvest, the element sinks reached a constant value (4,300 t for nitrogen sinks and 411 t for phosphorus sinks). The nitrogen and phosphorus sinks therefore greatly decreased after the non-harvest of *M. lutarioriparius* for 5 years compared to the harvest scenario.

## Discussion

### Amount of nitrogen and phosphorus fixed in plants in the field investigation

This study showed that element concentrations differed across plant organs. The concentrations of both nitrogen and phosphorus were the highest in the leaves, intermediate in the roots, and lowest in the stems, which was consistent with observations from previous studies on the wetland plant *P. australis* (Song and Shan, 2012). One possible reason for this may be that leaves are the main photosynthetic organ, with vigorous metabolism and high demand for nitrogen and phosphorus. Another possible reason could be that leaves can absorb some elements from water during prolonged flood periods (Bonanno and Giudice, 2010). Combined with the biomass of plant organs, the amount of nitrogen and phosphorus fixed in the three plant organs was the highest in the roots, intermediate in the stems, and lowest in the leaves. In Dongting Lake, the annual amount of nitrogen and phosphorus fixed by the aboveground parts of *M. lutarioriparius* reached 4,168 and 404 t, respectively. *M. lutarioriparius* demonstrated great potential for removing nitrogen and phosphorus from the lake as industrial raw materials.

Since elements in plants come from their environment, there is an obvious relationship between the contents of elements in the plants and their environment (Fernández-Aláez et al., 1999; Baldantoni et al., 2004; Chen et al., 2015). However, in this study, we found that the mean nitrogen and phosphorus concentrations in plants were not significantly correlated with their concentrations in soil. Kern-Hansen and Dawson (1978) also found that nutrient concentrations in plants had no obvious correlation with the concentrations in environments. The possible reasons for this may be as follows. Large amounts of nitrogen and phosphorus are deposited in the lake during the flood period from April to October in Dongting Lake, which changes the concentrations of nitrogen and phosphorus in soils and thus weakens the relationship between the element concentrations in plants and those in soils. On the other hand, during the flood period, plants can absorb nitrogen and phosphorus from the water, reducing the links between the element concentrations in plants and soils. Additionally, significant regression coefficients were observed between the mean phosphorus concentrations in plants with plot elevation, soil moisture, soil organic matter concentration, and soil total potassium in this study.

### Amounts of nitrogen and phosphorus released in the *in situ* experiment

In this study, the decomposition rate of stems was significantly lower than that of leaves. Similar results were obtained from a study on *Salix triandroides*, a native plant species in the Dongting Lake wetlands (Bian, 2020). Compared with leaves, stems and branches contain higher concentrations of lignin and cellulose, which contribute to the lower decomposition rate in these plant organs (dos Santos Fonseca et al., 2013). The stems, therefore, require a longer time to complete decomposition compared to the leaves (Xie et al., 2017). It was found that the half decomposition time was 1.12 years for stems and 0.61 years for leaves, and the complete decomposition time was 4.95 years for stems and 2.83 years for leaves. Unlike the stems on the soil surface designed in the *in situ* experiment, some stems in the field remained standing in the non-harvest scenario. These stems that remain standing are often colonized slowly by decomposing organisms (Mäkinen et al., 2006); hence, standing stems have a lower decomposition rate than that of fallen stems. Therefore, the half and complete decomposition time for the stems of *M. lutarioriparius* would be slightly longer than the estimated time.

The relative release indices changed obviously over time. For leaves, the relative release indices of nitrogen and phosphorus were above zero, indicating that there was a net release of nitrogen and phosphorus. This result is consistent with previous research conducted on the wetland plant *Carex cinerascens* (Zhang et al., 2020). Litters were buried during flooding, and many decomposition products were either absorbed on soil exchange surfaces or remained available for direct root uptake (Shure et al., 1986). For stems, the relative release indices of nitrogen and phosphorus were lower than zero in the middle stage of the experiment and higher than zero in the early and later stages, indicating a net fixation of nitrogen and phosphorus in the middle stage. Unlike other experiment conditions, the litter *in situ* experiment in this study was in a special environment condition with alternate flooding and non-flooding. During the long flood period, large amounts of nutrients, such as phosphorus, were carried by water into the lake and deposited into the sediment (Huang et al., 2022). Meanwhile, some nutrients were partially absorbed by the litter (Wang et al., 2018). Exogenous nitrogen and phosphorus supplementation, therefore, leads to increased concentrations of nitrogen and phosphorus in litters in the flood period (Figure 5) and contributes to negative relative release indices of the two elements in the period (Figure 3).

After litter decomposition of 1 year, the relative release indices of nitrogen and phosphorus in the leaves reached 87.2 and 78.4%, and those in the stems reached 39.7 and 48.6%, respectively. Combined with the distribution area of *M. lutarioriparius* in the Dongting Lake wetlands and the plant biomass in the unit area, the release amounts of nitrogen and phosphorus from the aboveground parts in the first year after the non-harvest of the plant were 2,394, and 232 t, respectively.

### Nitrogen and phosphorus sinks between harvest and non-harvest

In the harvest scenario, the aboveground parts of *M. lutarioriparius* were harvested and removed from the Dongting Lake wetlands, and annual element sinks were 4,168 t for nitrogen and 404 t for phosphorus. In the non-harvest scenario, considering that the aboveground parts were not harvested and would decompose in the wetland ecosystems, the element sinks in the first year after the non-harvest were 1,774 t for nitrogen and 172 t for phosphorus. Based on the model of mass residual ratio in litters in the *in situ* experiment, it was found that the nitrogen and phosphorus sinks increased greatly over time in the harvest scenario, while they increased gradually from the first year to the fifth year in the non-harvest scenario and reached a maximum value after the fifth year. Nitrogen and phosphorus sinks therefore greatly decreased after the non-harvest of *M. lutarioriparius* compared to that after harvest.

Besides nitrogen and phosphorus, plant harvest also removes several other pollutants, such as heavy metals. A study conducted by Yao et al. (2018) demonstrated that 0.7 t cadmium, 22.9 t copper, 77.5 t manganese, 3.1 t lead, and 95.9 t zinc were removed per year from the Dongting Lake wetlands through the annual harvest of the aboveground parts of *M. lutarioriparius*. Additionally, a large amount of the fixed elements (nitrogen, phosphorus, heavy metals, etc.) would be released into the environment, leading to new ecological and environmental problems in the non-harvest scenario. A new study demonstrated that the decomposition of *M. lutarioriparius* consumed the dissolved oxygen and increased the carbon, nitrogen, and phosphorus concentrations in the water (Zhao et al., 2021). The non-harvest of *M. lutarioriparius* may therefore have far-reaching impacts on the Dongting Lake wetlands. On the other hand, *M. lutarioriparius* is used to make diverse products, such as acetylated lignin, 2,5-furandicarboxylic acid, and particleboard (Chen et al., 2018; Chai et al., 2021; Liao et al., 2021). It is therefore recommended to continue to harvest *M. lutarioriparius* for ecosystem health and stability.

## Data availability statement

The raw data supporting the conclusions of this article will be made available by the authors, without undue reservation.

## Author contributions

YL designed the study. ZP and YD conducted the field investigation and the *in situ* experiment. SN, LX, and YN participated the two experiments. ZP and YL wrote the manuscript and other authors revised it. All authors contributed to the article and approved the submitted version.
