# Supplementary material for: Differences in nitrogen and phosphorus sinks between the harvest and non-harvest of Miscanthus lutarioriparius in the Dongting Lake wetlands
Source: Front Plant Sci. 2022 Sep 8;13:989931. doi: 10.3389/fpls.2022.989931 (PMC9493358; doi:10.3389/fpls.2022.989931)
Supplement: Supplementary file 1 [file Data_Sheet_1.PDF]

Attachment

Longitude and latitude of plots in the field investigation

Plots	Longitude (E)	Latitude (N)	Plots	Longitude (E)	Latitude (N)
1	113.0553	29.4453	13	113.0741	29.4136
2	113.0153	29.3851	14	113.0338	29.3942
3	113.0051	29.3764	15	113.0407	29.3968
4	113.0083	29.3609	16	113.0502	29.4046
5	112.7773	29.4612	17	112.8406	29.3899
6	112.7899	29.4578	18	112.7863	29.2724
7	112.8076	29.4308	19	112.7972	29.2806
8	112.8056	29.4341	20	112.7856	29.2572
9	112.8240	29.4359	21	112.7621	29.2232
10	112.8285	29.4344	22	112.7124	29.1211
11	112.8310	29.4208	23	112.7161	29.1238
12	113.0678	29.4179	24	112.7058	29.1079
